# Supplementary material for: Measures of Oxidative Status Markers in Relation to Age, Sex, and Season in Sick and Healthy Captive Asian Elephants in Thailand
Source: Animals (Basel). 2023 May 5;13(9):1548. doi: 10.3390/ani13091548 (PMC10177462; doi:10.3390/ani13091548)
Supplement: Supplementary file 1 [file animals-13-01548-s001.zip › animals-2268050-supplementary.pdf]

SUPPLEMENTARY DATA

Table S1. Data on age and sex of sick and selected healthy elephants in this study.

**Sick elephants (N=20)**		**Selected healthy (N=30)**
**Conditions**	**Age / sex**	**Age / sex**
EEHV-HD	1y / male	2y / male
EEHV-HD	2y / male	2y / male
EEHV-HD	4y / male	2y / male
		3y / male
		3y / male
		4y / male
EEHV-HD	3y / female	3y / female
EEHV-HD	4y / female	4y / female
MS	5y / female	4y / female
		4y / female
		5y / female
		5y / female
Eye	11y / female	9y / female
		10y / female
MS	14y / female	15y / female
GI	24y / female	23y / female
MS	31y / female	30y / female
GI	35y / female	33y / female
Wound	35y / female	37y / female
GI	40y / female	40y / female
		41y / female
MS	42y / female	42y / female
		42y / female
Eye	47y / female	46y / female
		46y / female
Eye	48y / female	48y / female
GI	50y / female	50y / female
GI	50y / female	53y / female
Wound	50y / female	54y / female
Weakness	55y / female	60y / female

y: years; EEHV-HD: Elephant endotheliotropic herpesvirus hemorrhagic disease; MS: musculoskeletal problem; GI: gastrointestinal problem.

Table S2. Mean (±SD) and ranges of biomarker concentrations in selected age-, sex- or camp-matched healthy elephants compared to all healthy elephants in the study.

**Biomarkers**	**Selected healthy elephants (N=30)**	**Other healthy elephants (N=107)**	**t statistic**	**P-value**
ROS (mg/l)	2.18 ± 0.14 (1.86-2.38)	2.18 ± 0.16 (1.61-2.53)	0.185	0.853
MDA (nmol/ml)	1.43 ± 0.35 (0.90-2.49)	1.78 ± 0.77 (0.75-3.28)	4.544	0.121
Albumin (g/dl)	3.23 ± 0.33 (2.70-3.90)	3.35 ± 0.36 (2.10-4.10)	1.665	0.098
GPx (U/l)	1.13 ± 0.47 (0.30-2.09)	1.16 ± 0.58 (0.23-3.04)	0.245	0.806
Catalase (U/ml)	13.25 ± 4.85 (6.33-25.64)	12.99 ± 5.73 (3.28-28.23)	-0.231	0.817
fGCM (ng/g)	55.43 ± 19.01 (22.15-103.23)	50.83 ± 17.15 (16.94-106.08)	-1.038	0.302

Table S3. Mean (± SD) and ranges of biomarker concentrations in sick and elephant endotheliotropic herpesvirus-hemorrhagic disease (EEHV-HD)-infected elephants compared to age-, sex- or camp-matched healthy elephants.

**Biomarkers**	**Reference range**	**Selected healthy elephants (n=30)[1]**	**Weakness (N=1)**	**Wound (N=2)**	**GI[2] (N=5)**	**Eye (N=3)**	**MS[3] (N=4)**	**EEHV-HD (N=5)**	**H statistic**	**P-value**
ROS (mg/l)	1.91 – 2.47	2.18 ± 0.14 (1.86-2.38)	2.24	1.92 ± 0.25 (1.72-2.08)	2.10 ± 0.15 (1.97-2.34)	2.11 ± 0.13 (1.97-2.24)	2.16 ± 0.15 (2.02-2.37)	2.19 ± 0.16 (1.97-2.38)	5.48	0.483
MDA (nmol/ml)	0.93 – 2.49	1.43 ± 0.35[a] (0.90-2.09)	1.49	1.87 ± 0.11 (1.79-1.94)	2.00 ± 0.57[ab] (1.34-2.84)	2.63 ± 1.50[ab] (1.49-4.33)	2.68 ± 1.06[ab] (1.79-3.88)	3.55 ± 0.29[b] (3.28-4.03)	24.44	<0.01**
Albumin (g/dl)	2.73 – 3.96	3.23 ± 0.33[a] (2.70-3.90)	1.10	3.45 ± 0.07 (3.40-3.50)	3.00 ± 0.26[ab] (2.70-3.30)	3.13 ± 0.21[ab] (2.90-3.30)	3.20 ± 0.26[ab] (3.00-3.50)	2.20 ± 0.29[b] (1.90-2.50)	16.14	0.013*
GPx (U/l)	0.14 – 2.08	1.13 ± 0.47 (0.30-2.09)	0.71	0.69 ± 0.94 (0.03-1.36)	1.04 ± 0.23 (0.80-1.32)	0.86 ± 0.21 (0.65-1.07)	0.87 ± 0.52 (0.50-1.64)	1.76 ± 0.40 (1.24-2.25)	10.92	0.091
Catalase (U/ml)	2.99 – 21.86	13.25 ± 4.85 (6.33-25.64)	12.02	7.56 ± 3.43 (5.13-9.99)	16.73 ± 7.52 (6.01-25.51)	15.28 ± 5.53 (11.29-21.61)	14.67 ± 1.29 (12.76-15.57)	23.81 ± 10.76 (12.52-41.78)	10.71	0.098
fGCM[4] (ng/g)	22.23 – 82.70	55.43 ± 19.01 (22.15-79.40)	273.86	55.69 ± 5.64 (51.70-59.68)	95.14 ± 53.81 (32.30-161.11)	36.09 ± 1.69 (34.90-37.28)	51.64 ± 26.68 (32.78-70.51)	42.00 ± 13.63 (26.48-52.03)	8.79	0.185

[a,b]Different superscripts indicate a significant difference at P<0.05. Weakness and Wound categories were excluded from the analysis due to low N numbers.

[1]Age-, sex- and camp-matched

[2]Gastrointestinal system

[3]Musculoskeletal system

[4]Fecal glucocorticoid metabolites

Asterisks indicated the significant levels at $p < 0.05$ (*) and $p < 0.01$ (**)

Table S4. Generalized linear model analyses of variables associated with serum reactive oxygen species (ROS) concentrations.

**Variables**		**N**	**Estimate**	**SE**	**t value**	**P**
	(intercept)		2.207	0.049	44.914	<0.01**
Age group	Juvenile	45	(Reference)			
	Subadult	36	0.109	0.085	1.282	0.202
	Adult	45	0.514	0.200	2.564	0.011*
	Aged	11	0.300	0.138	2.158	0.032*
Sex	Male	45	(Reference)			
	Female	92	-0.148	0.109	-1.348	0.180
Season	Summer	33	(Reference)			
	Rainy	53	-0.104	0.062	-1.663	0.098
	Winter	51	0.023	0.094	0.250	0.803
Age group x Sex	Juvenile x Male	24	(Reference)			
	Subadult x Female	23	0.163	0.144	1.133	0.259
	Adult x Female	37	-0.221	0.170	-1.302	0.195
	Aged x Female	11	N/A	N/A	N/A	N/A
Age group x Season	Juvenile x Summer	10	(Reference)			
	Subadult x Rainy	22	-0.169	0.107	-1.577	0.117
	Adult x Rainy	-	N/A	N/A	N/A	N/A
	Aged x Rainy	-	N/A	N/A	N/A	N/A
	Subadult x Winter	5	-0.064	0.152	-0.424	0.672
	Adult x Winter	33	-0.556	0.177	-3.145	0.002**
	Aged x Winter	9	-0.509	0.202	-2.524	0.013*
Sex x Season	Male x Summer	12	(Reference)			
	Female x Rainy	33	0.143	0.121	1.185	0.238
	Female x Winter	38	0.343	0.194	1.766	0.080
Age groups x Sex x Season	Juvenile x Male x Summer	8	(Reference)			
	Subadult x Female x Rainy	15	-0.047	0.165	-0.285	0.776
	Adult x Female x Rainy	-	N/A	N/A	N/A	N/A
	Aged x Female x Rainy	-	N/A	N/A	N/A	N/A
	Subadult x Female x Winter	3	-0.422	0.250	-1.687	0.094
	Adult x Female x Winter	25	N/A	N/A	N/A	N/A
	Aged x Female x Winter	9	N/A	N/A	N/A	N/A

Asterisks indicated the significant levels at $p < 0.05$ (*) and $p < 0.01$ (**).

Table S5. Generalized linear model analyses of variables associated with serum malondialdehyde (MDA) concentrations.

Variables		N	Estimate	SE	t value	P
	(intercept)		1.998	0.156	12.819	<0.01**
Age group	Juvenile	45	(Reference)			
	Subadult	36	-0.321	0.270	-1.190	0.236
	Adult	45	-0.894	0.636	-1.407	0.162
	Aged	11	-0.600	0.441	-1.361	0.176
Sex	Male	45	(Reference)			
	Female	92	0.391	0.348	1.122	0.264
Season	Summer	33	(Reference)			
	Rainy	53	-0.162	0.198	-0.820	0.413
	Winter	51	0.091	0.298	0.306	0.760
Age group x Sex	Juvenile x Male	24	(Reference)			
	Subadult x Female	23	0.081	0.457	0.178	0.859
	Adult x Female	37	0.244	0.539	0.454	0.651
	Aged x Female	11	N/A	N/A	N/A	N/A
Age group x Season	Juvenile x Summer	10	(Reference)			
	Subadult x Rainy	22	0.532	0.340	1.565	0.120
	Adult x Rainy	-	N/A	N/A	N/A	N/A
	Aged x Rainy	-	N/A	N/A	N/A	N/A
	Subadult x Winter	5	0.471	0.485	0.972	0.333
	Adult x Winter	33	0.501	0.562	0.892	0.374
	Aged x Winter	9	0.550	0.641	0.858	0.392
Sex x Season	Male x Summer	12	(Reference)			
	Female x Rainy	33	-0.712	0.384	-1.856	0.065
	Female x Winter	38	-0.841	0.617	-1.363	0.175
Age groups x Sex x Season	Juvenile x Male x Summer	8	(Reference)			
	Subadult x Female x Rainy	15	0.093	0.524	0.177	0.860
	Adult x Female x Rainy	-	N/A	N/A	N/A	N/A
	Aged x Female x Rainy	-	N/A	N/A	N/A	N/A
	Subadult x Female x Winter	3	0.318	0.794	0.401	0.688
	Adult x Female x Winter	25	N/A	N/A	N/A	N/A
	Aged x Female x Winter	9	N/A	N/A	N/A	N/A

Asterisks indicated the significant levels at $p < 0.05$ (*) and $p < 0.01$ (**).

Table S6. Generalized linear model analyses of variables associated with serum albumin concentrations.

Variables		N	Estimate	SE	t value	P
	(intercept)		3.187	0.119	26.794	<0.01**
Age group	Juvenile	45	(Reference)			
	Subadult	36	0.287	0.206	1.395	0.165
	Adult	45	0.485	0.485	1.000	0.319
	Aged	11	0.850	0.336	2.526	0.012*
Sex	Male	45	(Reference)			
	Female	92	-0.287	0.266	-1.081	0.282
Season	Summer	33	(Reference)			
	Rainy	53	0.112	0.151	0.744	0.458
	Winter	51	0.212	0.227	0.933	0.353
Age group x Sex	Juvenile x Male	24	(Reference)			
	Subadult x Female	23	0.192	0.348	0.552	0.582
	Adult x Female	37	0.014	0.412	0.035	0.972
	Aged x Female	11	N/A	N/A	N/A	N/A
Age group x Season	Juvenile x Summer	10	(Reference)			
	Subadult x Rainy	22	-0.116	0.259	-0.447	0.655
	Adult x Rainy	-	N/A	N/A	N/A	N/A
	Aged x Rainy	-	N/A	N/A	N/A	N/A
	Subadult x Winter	5	0.162	0.369	0.439	0.661
	Adult x Winter	33	-0.348	0.428	-0.812	0.418
	Aged x Winter	9	-0.361	0.489	-0.739	0.461
Sex x Season	Male x Summer	12	(Reference)			
	Female x Rainy	33	0.171	0.293	0.583	0.561
	Female x Winter	38	-0.112	0.471	-0.239	0.811
Age groups x Sex x Season	Juvenile x Male x Summer	8	(Reference)			
	Subadult x Female x Rainy	15	-0.107	0.400	-0.268	0.789
	Adult x Female x Rainy	-	N/A	N/A	N/A	N/A
	Aged x Female x Rainy	-	N/A	N/A	N/A	N/A
	Subadult x Female x Winter	3	-0.409	0.606	-0.675	0.501
	Adult x Female x Winter	25	N/A	N/A	N/A	N/A
	Aged x Female x Winter	9	N/A	N/A	N/A	N/A

Asterisks indicated the significant levels at $p < 0.05$ (*) and $p < 0.01$ (**).

Table S7. Generalized linear model analyses of variables associated with serum glutathione peroxidase activity.

Variables		N	Estimate	SE	t value	P
	(intercept)		1.123	0.189	5.930	<0.01**
Age group	Juvenile	45	(Reference)			
	Subadult	36	-0.059	0.328	-0.179	0.858
	Adult	45	-0.464	0.773	-0.600	0.549
	Aged	11	-0.173	0.536	-0.324	0.747
Sex	Male	45	(Reference)			
	Female	92	0.656	0.423	1.550	0.124
Season	Summer	33	(Reference)			
	Rainy	53	0.236	0.241	0.983	0.328
	Winter	51	-0.425	0.363	-1.173	0.243
Age group x Sex	Juvenile x Male	24	(Reference)			
	Subadult x Female	23	-0.251	0.556	-0.452	0.652
	Adult x Female	37	-0.036	0.656	-0.055	0.956
	Aged x Female	11	N/A	N/A	N/A	N/A
Age group x Season	Juvenile x Summer	10	(Reference)			
	Subadult x Rainy	22	-0.429	0.413	-1.039	0.301
	Adult x Rainy	-	N/A	N/A	N/A	N/A
	Aged x Rainy	-	N/A	N/A	N/A	N/A
	Subadult x Winter	5	0.527	0.589	0.895	0.372
	Adult x Winter	33	0.711	0.683	1.041	0.299
	Aged x Winter	9	0.272	0.778	0.350	0.727
Sex x Season	Male x Summer	12	(Reference)			
	Female x Rainy	33	-0.948	0.466	-2.034	0.044*
	Female x Winter	38	-0.546	0.749	-0.729	0.467
Age groups x Sex x Season	Juvenile x Male x Summer	8	(Reference)			
	Subadult x Female x Rainy	15	1.214	0.638	1.904	0.059
	Adult x Female x Rainy	-	N/A	N/A	N/A	N/A
	Aged x Female x Rainy	-	N/A	N/A	N/A	N/A
	Subadult x Female x Winter	3	-0.012	0.965	-0.013	0.989
	Adult x Female x Winter	25	N/A	N/A	N/A	N/A
	Aged x Female x Winter	9	N/A	N/A	N/A	N/A

Asterisks indicated the significant levels at $p < 0.05$ (*) and $p < 0.01$ (**).

Table S8. Generalized linear model analyses of variables associated with serum catalase activity.

Variables		N	Estimate	SE	t value	P
	(intercept)		10.239	1.674	6.117	<0.01**
Age group	Juvenile	45	(Reference)			
	Subadult	36	-2.913	2.899	-1.005	0.317
	Adult	45	-3.538	6.831	-0.518	0.605
	Aged	11	-5.525	4.735	-1.167	0.245
Sex	Male	45	(Reference)			
	Female	92	2.457	3.743	0.656	0.513
Season	Summer	33	(Reference)			
	Rainy	53	2.041	2.127	0.960	0.339
	Winter	51	2.568	3.205	0.801	0.424
Age group x Sex	Juvenile x Male	24	(Reference)			
	Subadult x Female	23	2.052	4.909	0.418	0.676
	Adult x Female	37	-2.030	5.795	-0.350	0.727
	Aged x Female	11	N/A	N/A	N/A	N/A
Age group x Season	Juvenile x Summer	10	(Reference)			
	Subadult x Rainy	22	3.924	3.651	1.075	0.284
	Adult x Rainy	-	N/A	N/A	N/A	N/A
	Aged x Rainy	-	N/A	N/A	N/A	N/A
	Subadult x Winter	5	14.642	5.204	2.813	<0.01**
	Adult x Winter	33	9.188	6.032	1.523	0.130
	Aged x Winter	9	7.317	6.879	1.064	0.289
Sex x Season	Male x Summer	12	(Reference)			
	Female x Rainy	33	-3.105	4.121	-0.754	0.452
	Female x Winter	38	-3.098	6.625	-0.468	0.641
Age groups x Sex x Season	Juvenile x Male x Summer	8	(Reference)			
	Subadult x Female x Rainy	15	0.212	5.636	0.038	0.969
	Adult x Female x Rainy	-	N/A	N/A	N/A	N/A
	Aged x Female x Rainy	-	N/A	N/A	N/A	N/A
	Subadult x Female x Winter	3	-13.153	8.524	-1.543	0.125
	Adult x Female x Winter	25	N/A	N/A	N/A	N/A
	Aged x Female x Winter	9	N/A	N/A	N/A	N/A

Asterisks indicated the significant levels at $p < 0.05$ (*) and $p < 0.01$ (**).

Table S9. Generalized linear model analyses of variables associated with fGCM concentrations.

Variables		N	Estimate	SE	t value	P
	(intercept)		36.075	8.786	4.106	<0.01**
Age group	Juvenile	17	(Reference)			
	Subadult	16	4.984	13.421	0.371	0.711
	Adult	41	-32.551	28.462	-1.144	0.257
	Aged	11	-18.894	36.581	-0.517	0.607
Sex	Male	22	(Reference)			
	Female	63	29.492	33.265	0.887	0.378
Season	Summer	21	(Reference)			
	Rainy	16	12.466	19.647	0.635	0.528
	Winter	48	23.740	15.218	1.560	0.123
Age group x Sex	Juvenile x Male	7	(Reference)			
	Subadult x Female	9	-5.461	30.437	-0.179	0.858
	Adult x Female	33	6.379	22.675	0.281	0.779
	Aged x Female	11	N/A	N/A	N/A	N/A
Age group x Season	Juvenile x Summer	4	(Reference)			
	Subadult x Rainy	6	11.061	25.364	0.436	0.664
	Adult x Rainy	-	N/A	N/A	N/A	N/A
	Aged x Rainy	-	N/A	N/A	N/A	N/A
	Subadult x Winter	3	-9.198	22.112	-0.416	0.679
	Adult x Winter	33	30.336	30.428	0.997	0.322
	Aged x Winter	9	20.511	32.614	0.629	0.531
Sex x Season	Male x Summer	7	(Reference)			
	Female x Rainy	13	-19.681	38.074	-0.517	0.607
	Female x Winter	36	-40.665	25.364	-1.603	0.113
Age groups x Sex x Season	Juvenile x Male x Summer	4	(Reference)			
	Subadult x Female x Rainy	4	-12.299	38.744	-0.317	0.752
	Adult x Female x Rainy	-	N/A	N/A	N/A	N/A
	Aged x Female x Rainy	-	N/A	N/A	N/A	N/A
	Subadult x Female x Winter	1	N/A	N/A	N/A	N/A
	Adult x Female x Winter	25	N/A	N/A	N/A	N/A
	Aged x Female x Winter	9	N/A	N/A	N/A	N/A

Asterisks indicated the significant levels at $p < 0.05$ (*) and $p < 0.01$ (**).

Table S10. Mean (± SD) and ranges of blood parameters in sick elephants in different disease categories as compared to the reference ranges for healthy elephants.

Parameter		Reference Range (N=137)	Disease conditions (N=20)					
			Weakness (N=1)	Wound (N=2)	Gastrointestinal (N=5)	Eye (N=3)	Musculoskeletal (N=4)	EEHV-HD (N=5)
**RBC**	PCV (%)	27.62 - 47.52	27	34.50 ± 0.71 (34-35)	32.45 ± 1.71 (30-34)	33.50 ± 2.12 (32-35)	33.67 ± 0.58 (33-34)	35.80 ± 7.56 (26-44)
	Hemoglobin (g/dl)	10.29 – 16.10	9.9	12.55 ± 0.49 (12.20-12.90)	11.65 ± 0.42 (11.20-12.10)	11.85 ± 0.35 (11.60-12.10)	12.40 ± 0.10 (12.30-12.50)	12.34 ± 3.06 (9.20-14.80)
	RBC count (X 10^6 cells/μl)	2.39 – 3.91	2.23	2.75 ± 0.04 (2.72-2.78)	2.69 ± 0.17 (2.49-2.71)	2.70 ± 0.13 (2.61-2.80)	2.84 ± 0.14 (2.75-3.00)	3.24 ± 0.65 (2.87-3.97)
	MCV (fl)	106.30 – 129.08	122.8	126.00 ± 0.42 (125.70-126.30)	120.70 ± 3.49 (115.50-123.00)	123.55 ± 1.48 (122.50-124.60)	119.17 ± 4.46 (114.50-123.40)	110.98 ± 3.43 (105.20-114.30)
	MCHC (g/dl)	34.62 – 36.82	36.1	36.30 ± 0.85 (35.70-36.90)	36.05 ± 1.24 (34.70-37.70)	35.50 ± 1.13 (34.70-36.30)	36.67 ± 0.42 (36.20-37.00)	34.20 ± 2.83 (30.50-36.40)
**WBC**	WBC count (cells/μl)	7,130.58 – 20,368.19	12,580	10,100.00 ± 282.84 (9,900-10,300)	14,602.50 ± 5,776.03 (8,680-21,630)	14,180 ± 5,289.16 (10,440-17,920)	10,020 ± 4,309.65 (5,170-13,410)	18,950.00 ± 11,131.23 (9,700-36,320)
	Heterophil (cells/μl)	974.61 – 5,383.52	5,787	2,687.50 ± 860.55 (2,079-3,296)	4,211.25 ± 2,812.71 (1,389-7,595)	3,984.50 ± 566.39 (3,584-4,385)	2,698.00 ± 1,746.86 (1,448-4,694)	9,502.80 ± 4,755.95 (5,044-15,254)
	Lymphocyte (cells/μl)	686.23 – 9,985.99	2,893	3,693.00 ± 3,037.73 (1,545-5,841)	4,588.00 ± 851.56 (3,506-5,468)	5,232.00 ± 2,231.63 (3,654-6,810)	3,086.00 ± 1,576.15 (1,448-4,592)	5,909.40 ± 2,778.33 (3,502-10,170)
	Monocyte (cells/μl)	422.91 – 7,459.65	3,774	3,608.50 ± 2,443.05 (1,881-5,336)	4,393.00 ± 2,192.23 (1,649-6,884)	4,307.00 ± 3,285.22 (1,984-6,630)	4,057.33 ± 1,544.47 (2,274-4,962)	3,351.40 ± 4,078.38 (721-10,533)
	Eosinophil (cells/μl)	0 – 598.60	126	101.00 ± 2.83 (99-103)	1,335.75 ± 2,292.98 (168-4,775)	657.00 ± 337.99 (418-896)	178.67 ± 309.46 (0-536)	186.40 ± 215.53 (103-466)
	Basophil (cells/μl)	0 – 100.33	Not found	Not found	74.50 ± 149.00 (0-298)	Not found	Not found	Not found
	H:L ratio	>0 – 1.47	2.00	1.24 ± 1.26 (0.36-2.13)	0.93 ± 0.56 (0.25-1.51)	0.86 ± 0.48 (0.53-1.20)	0.96 ± 0.52 (0.42-1.46)	1.59 ± 0.26 (1.37-2.00)
	M:H ratio	>0 – 2.73	0.65	1.26 ± 0.50 (0.90-1.62)	1.26 ± 0.62 (0.52-2.04)	1.15 ± 0.99 (0.45-1.85)	1.72 ± 0.75 (1.05-2.53)	0.30 ± 0.24 (0.12-0.69)
**Platelet**	Platelet count (X 10^3 cells/μl)	151.08 – 552.62	407	405.50 ± 51.62 (369-442)	378.25 ± 64.08 (321-459)	406.50 ± 153.44 (298-515)	419.67 ± 52.08 (366-470)	118.40 ± 103.71 (22-143)
**Blood chemistry**	BUN (mg/dl)	4.44 – 15.31	14	7.35 ± 1.48	9.27 ± 2.93 (6.6-12.0)	9.10 ± 2.75 (6.1-12.0)	6.50 ± 0.98 (5.4-7.3)	12.80 ± 2.00 (11.0-16.0)

			(6.3-8.4)				
Creatinine (mg/dl)	0.90 – 1.89	1.57	1.11 ± 0.11 (1.04-1.19)	1.39 ± 0.57 (0.91-2.11)	1.28 ± 0.14 (1.14-1.42)	1.41 ± 0.54 (1.02-2.03)	2.20 ± 0.33 (1.79-2.54)
ALT (U/L)	0 – 2.11	37	1.50 ± 2.12 (0-3)	0.50 ± 0.57 (0-1)	1.83 ± 0.57 (2-3)	1.67 ± 0.58 (1-2)	5.75 ± 3.59 (3-11)
ALP (U/L)	0 – 306.92	29	88.00 ± 14.14 (78-98)	82.80 ± 34.35 (49-94)	83.67 ± 40.08 (50-128)	160.50 ± 122.15 (75-335)	113.00 ± 28.08 (73-131)
Total serum protein (g/dl)	6.81 – 9.91	8.3	8.65 ± 0.64 (3.4-3.5)	8.32 ± 0.87 (7.4-9.4)	8.30 ± 0.80 (7.5-9.1)	7.17 ± 0.50 (6.7-7.7)	5.57 ± 0.76 (5-6)

PCV: packed cell volume; RBC: red blood cells; MCV: mean corpuscular volume; MCHC: mean corpuscular hemoglobin concentrations; WBC: total white blood cells; H:L ratio: Heterophil to Lymphocyte ratio; M:H ratio: Monocyte to Heterophil ratio; BUN: blood urea nitrogen; ALT: alanine transaminase; ALP: alanine phosphatase.

Table S11. Biomarker concentrations (mean ± SD) and statistical comparisons between calves that survived or succumbed to EEHV-HD using the Mann-Whitney U tests.

Biomarkers	Survived (N=2)	Died (N=3)	U statistic	P-value
ROS (mg/l)	2.31 ± 0.09	2.11 ± 0.15	1	0.386
MDA (nmol/ml)	3.36 ± 0.11	3.68 ± 0.31	5.5	0.236
Albumin (g/dl)	2.25 ± 0.35	2.15 ± 0.35	1	0.698
GPx (U/l)	1.53 ± 0.41	1.92 ± 0.39	5	0.386
Catalase (U/ml)	17.36 ± 6.85	28.29 ± 11.69	4	0.773
fGCM (ng/g)	52.03 (N=1)	36.98 ± 14.86	0	0.540
